# Repeated centrifuging and washing concentrates bacterial samples in peritoneal dialysis for optimal culture: an original article

**DOI:** 10.1186/s12866-020-02044-7

**Published:** 2020-11-27

**Authors:** Ni Tien, Bang-Jau You, Hsuan-Jen Lin, Chieh-Ying Chang, Che-Yi Chou, Hsiu-Shen Lin, Chiz-Tzung Chang, Charles C. N. Wang, Hung-Chih Chen

**Affiliations:** 1 Department of Laboratory Medicine, China Medical University Hospital, Taichung, Taiwan; 2 Department of Medical Laboratory Science and Biotechnology, China Medical University, Taichung, Taiwan; 3 Department of Chinese Pharmaceutical Sciences and Chinese Medicine Resources, Taichung, Taiwan; 4 China Medical University, Taichung, Taiwan; 5 Division of Nephrology, Asia University Hospital, Taichung, Taiwan; 6 College of Medicine, Kaohsiung Medical University, Kaohsiung, Taiwan; 7 College of Medicine, China Medical University, Taichung, Taiwan; 8 Division of Nephrology, China Medical University Hospital, No. 2, Yu-der Road, North District, Taichung, 40447 Taiwan; 9 Department of Bioinformatics and Medical Engineering Asia University, Taichung, 41354 Taiwan; 10 Center for Artificial Intelligence and Precision Medicine Research, Asia University, 500, Lioufeng Rd., Wufeng, Taichung, Taiwan; 11 Division of Nephrology, Asia University Hospital, No. 222, Fuxin Road, Wufeng District, Taichung, 41354 Taiwan

**Keywords:** Repeat centrifuging and washing, Peritonitis, Peritoneal dialysis, Bacterial culture

## Abstract

**Background:**

Bacterial cultures allow the identification of infectious disease pathogens. However, obtaining the results of conventional culture methods is time-consuming, taking at least two days. A more efficient alternative is the use of concentrated bacterial samples to accelerate culture growth. Our study focuses on the development of a high-yield sample concentrating technique.

**Results:**

A total of 71 paired samples were obtained from patients on peritoneal dialysis (PD). The peritoneal dialysates were repeat-centrifuged and then washed with saline, namely the centrifuging and washing method (C&W method). The concentrated samples were Gram-stained and inoculated into culture plates. The equivalent unprocessed dialysates were cultured as the reference method. The times until culture results for the two methods were compared. The reference method yielded no positive Gram stain results, but the C&W method immediately gave positive Gram stain results for 28 samples (*p* < 0.001). The culture-negative rate was lower in the C&W method (5/71) than in the reference method (13/71) (*p* = 0.044). The average time for bacterial identification achieved with the C&W method (22.0 h) was shorter compared to using the reference method (72.5 h) (*p* < 0.001).

**Conclusions:**

The C&W method successfully concentrated bacterial samples and superseded blood culture bottles for developing adequate bacterial cultures. The C&W method may decrease the culture report time, thus improving the treatment of infectious diseases.

## Background

The current method for culturing samples of infected body fluids involves inoculating the specimen into a blood culture bottle and then incubating the sample within an automated culture machine [1, 2]. After the machine automatically detects bacterial signals within the blood culture bottle, the broth within the blood culture bottle is streaked onto culture plates containing different culture media for further culture. Biochemical methods can identify bacterial colonies, which are subsequently isolated. One example of such a method is matrix-assisted laser desorption/ionization time-of-flight mass spectrometry (MALDI-TOF MS) [3–5]. After bacterial identification, the antibiotic susceptibility test is performed using the disc diffusion of automated antimicrobial susceptibility testing systems [6]. This conventional bacterial culture procedure is time-consuming, with a minimum of 2 days for obtaining the final report. When treating infectious diseases in humans, empirical antibiotics are prescribed immediately for typical pathogen coverage and are then adjusted to the most effective agents based on the final reports [7]. The bacterial growth rate depends on both the physical and chemical conditions of the culture medium. The number of bacteria added to the culture medium can also affect the bacterial growth rate. A concentrated sample can accelerate the bacterial growth rate [8]. Our study uses repeat centrifuging and washing (C&W method) for the infected peritoneal dialysate culture. The method for specimens was paired with the conventional method as the reference method. The times until culture results for the two methods were compared.

## Results

### Positive and negative control groups

**Fig. 1:**
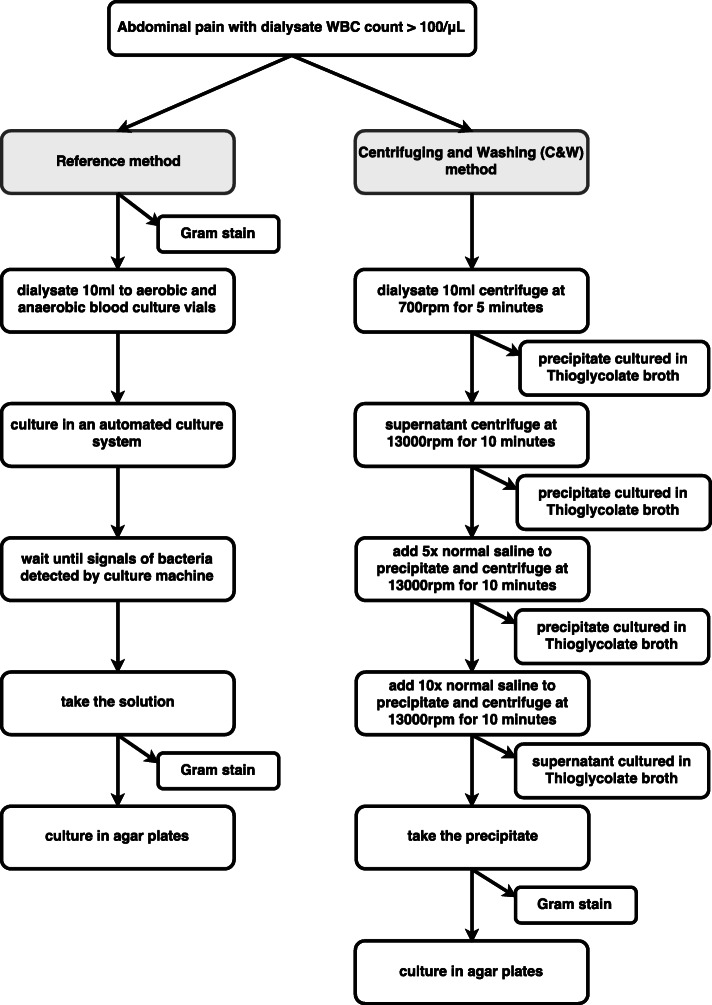
The diagram to report the participants and culture results through the study

Twenty-four patients receiving peritoneal dialysis (PD) with no abdominal-pain donated clear dialysate for control samples. A total of 12 clear dialysates were used as negative controls (no bacteria were added), and these samples were subjected to the C&W method of culturing. All 12 samples had culture-negative results. Another 12 clear dialysate samples were divided equally into two groups for the positive controls. Group 1 samples (*n* = 6) were inoculated with *Escherichia coli (E. coli)* (American Type Culture Collection (ATCC) 25,922), and Group 2 samples (*n* = 6) were inoculated with *Staphylococcus aureus (S. aureus)* (ATCC 25923). These samples were also subjected to C&W processing and cultured. The bacteria in each sample were identified as the species added to the sample before being cultured (Fig. 1).

In the six experiments that used *E. coli* (ATCC 25922) for culture, the time required to obtain the Gram stain reports for the reference method was 238 ± 65 min. In contrast, the average Gram stain report time for samples processed using the C&W method was 40 ± 0 min, which was the sum of the time needed to perform the C&W and Gram stain. The Gram-negative bacilli *(E. coli)* results were received before sending the concentrated samples for further culture. The time needed for the Gram stain results to be reported for the C&W method was shorter than the reference method (*p* < 0.001) (Table 1). The median number of colony-forming units (CFU) for the un-processed samples seeded on agar plates was 5.6 × 10^4^ (interquartile range, IQR: 3.8 × 10^4^–6.9 × 10^4^) CFU/mL, while the median number of CFU for the C&W method was 10 × 10^4^ (IQR: 8.7 × 10^4^–10.0 × 10^4^) CFU/mL. The median CFU number was higher in the C&W processed samples (*p* = 0.015) than the conventionally processed samples (Table 1). The ATP generation test revealed that bacteria from unconcentrated samples emitted a signal in the form of relative light units (RLU) (27.6 ± 3.6 RLU). The ATP generation signal was found to be significantly weaker than that from the C&W processed samples (49.1 ± 6.6 RLU) (*p* < 0.001) (Table 1).

**Table 1:** The results of culture report times and ATP test for *E. coli* (ATCC 25922) cultured by reference or C&W methods

	Reference	C&W	***p***-value
Gram stain report time (minutes)	238 ± 65	40 ± 0	< 0.001
Colony count*(×10^4^ CFU/ml)	5.6 (3.8–6.9)	10 (8.7–10)	0.015
ATP generation test (RLU^#^)	27.6 ± 3.6	49.1 ± 6.6	< 0.001

*C&W* centrifuging and washing method, ^*#*^ *RLU* relative light unit

* colony count > 10^5^ CFU/ml was counted as 10^5^ CFU/ml

### Positive/negative controls and peritonitis patients

In addition to the 12 samples used as positive controls and 12 used as negative controls, a further 51 patients receiving PD with peritonitis donated dialysate samples. Four patients were excluded because of sample errors (Fig. 1).

For all 71 paired dialysate samples, the mean dialysate white blood cells (WBC) count was 2722 ± 414 cells/μL, of which 82.1% ± 13.1% were polymorphonuclear. Of the 71 samples, 58 had culture-positive (CP) results, and 13 had culture-negative (CN) results when using the reference method. For the C&W method, 66 samples had a CP result, and 5 had a CN result. The CN rate was lower for the C&W method than for the reference method. The C&W method obtained the same results as the reference method for 93.1% (54 of the 58) of the CP samples in pathogen identification. The two methods obtained different pathogen identification results in three patients (Table 2).

**Table 2:** Demographic data and culture results of peritoneal dialysis patients

Patient number (***n***)	71
Dialysate white blood cell count (/μL)	2722 ± 414
Dialysate polymorphonuclear cells (%)	82.1 ± 13.1
CP:CN (Reference method) (*n*)	58:13
CP:CN (C&W method) (*n*)	66: 5
Double negative by two methods (%, *n*)	5.6% (4/71)
Same pathogens in CP by both methods (%, *n*)	93.1% (54/58)
Different pathogens in CP by both methods (%, *n*)	4.2% (3/71)

*C&W* method: centrifuging and washing method, *CP* culture-positive, *CN* culture-negative

Table 3 lists the four samples that were CP with the reference method. However, when using the C&W method, different results were obtained. Nine out of the 13 samples that were CN with the reference method were reported as CP with the C&W method. The remaining CN samples were also CN with the C&W method. In terms of patient outcomes, 5 of the 17 patients listed in Table 3 were not cured, and their PD catheters were removed. All five of these patients had different results reported by the two methods. The C&W method reported fungi in three of the five results-mismatched patients (Table 3).

**Table 3:** The different culture results of the reference method and C&W method

Case	Reference method	C&W method	Outcome
1	No growth	*Candida albicans*	Catheter removal
2	No growth	*Staphylococcus capitis*	Cured
3	No growth	*OSSA*	Cured
4	No growth	*Staphylococcus epidermititis*	Catheter removal
5	No growth	*Staphylococcus epidermititis*	Cured
6	No growth	*Staphylococcus capitis*	Cured
7	No growth	*Hemophilus influenza*	Cured
8	No growth	*Escherichia coli*	Cured
9	No growth	*Staphylococcus epidermititis*	Cured
10	No growth	No growth	Cured
11	No growth	No growth	Cured
12	No growth	No growth	Cured
13	No growth	No growth	Cured
14	*Klebsiella pneumonia*	No growth	Cured
15	Gram-positive bacilli	*Aspergillus fumigatus*	Catheter removal
16	*Klebsiella oxytoca*	*Roulette ornithinolytica*	Catheter removal
17	*P. aeruginosa*	*Candida spp.*	Catheter removal

*C&W method* centrifuging and washing method, *OSSA oxacillin-sensitive Staphylococcus aureus, P. aeruginosa: Pseudomonas aeruginosa*

### The culture result report times of the two different methods

No pathogens were found by routine Gram staining in any 71 pre-culture samples in the reference group. However, Gram staining identified pathogens in 28 samples after the centrifuging and washing treatment but before streaking the samples on agar plates for subsequent culture (*p* < 0.001). The lengths of times needed for pathogens to be identified or for antibiotic susceptibility test results to be available were significantly shorter for the C&W method compared with the reference method. The trends were similar for all 71 pairs of samples and the 54 paired-samples for which the same culture results were obtained using both methods (Table 4).

**Table 4:** The comparisons of the consuming time by the different methods

	Reference	C&W method	***p***
**All patients (*n*** **= 71)**			
Results before samples cultured	0	28	< 0.001
Bacteria identification time (hours)	72.5 (41.5–123.5)	22.0 (15.0–40.0)	< 0.001
Sensitivity report time (hours)	92.0 (71.0–144.0)	37.0 (30.0–55.0)	< 0.001
**Same culture results patients (*n*** **= 54)**			
Bacteria identification time (hours)	59.0 (33.5–79.7)	21.0 (14.0–24.0)	< 0.001
Sensitivity report time (hours)	89.5 (68.2–102.5)	35.0 (28.2–40.0)	< 0.001

*C&W method* centrifuging and washing method

### Potential benefits of the C&W method

As this was a diagnostic accuracy study, we did not guide our treatment based on the C&W method results. Faster bacteria identification by the C&W method may have potential benefits. For the 54 paired-samples for which the same results were obtained using both methods, the culture report time (including bacterial identification and antibiotic-sensitivity report) was shorter (Table 5) for the C&W method. It meant that vancomycin could have been used earlier in five patients with methicillin-resistant *Staphylococcus aureus* (*MRSA*) infection. Likewise, gentamicin could have been stopped earlier in the 31 patients identified with Gram-positive bacterial infection.

**Table 5:** The shortened reporting time and early antibiotics adjusting by C&W method

Shortened time	Same culture results (***n*** = 54)	All results (***n*** = 71)
Bacteria identification (hours)	38.6 ± 31.2	43.3 ± 40.1
Antibiotic sensitivity report (hours)	51.7 ± 31.6	49.1 ± 42.4
Early Vancomycin use (*n*)	5	6
Early Gentamicin quit (*n*)	31	36
Early peritoneal catheter removal (*n*)	NA	3

*C&W method* centrifuging and washing method

Similarly, when all the 71 pair-samples were included, the C&W method shortened the culture report times. Vancomycin usage and gentamicin withdrawal could have taken place at a sooner time if decisions were based on the result of the C&W method. Patients with fungal peritonitis can also have their PD catheter removed earlier (Table 5).

## Discussion

The bacterial infection is the most common cause of PD-associated peritonitis and, in turn, the most common cause of peritoneal technique failure and patient mortality [9]. A quick and accurate pathogen diagnosis facilitates treatment. Although previous studies have demonstrated that sample centrifugation can increase bacterial concentration [10–13], sample washing was not used in these studies. These studies focused on food-borne pathogens, tuberculosis, parasites, and meningitis. Centrifuging 50 mL of PD fluid before culturing PD peritonitis is not practical for clinical practice, although it has been suggested for replacing the culture method without centrifuging [7]. The typical syringe used for PD fluid or ascites sampling is a 10-mL or 20-mL syringe. The process of sampling 50 mL of PD fluid and transporting it to a bacterial laboratory is inconvenient.

Furthermore, samples are often not processed immediately during non-office hours. Another concern is the possible contamination during centrifuging. Therefore, bedside inoculation of 10 mL ascites fluid into a tryptic soy broth (TSB) blood culture bottle has replaced the conventional method of using chocolate agar, blood agar, MacConkay agar, or thioglycolate broth as standard culture media for spontaneous bacterial peritonitis [14]. We used a bedside blood culture bottle inoculating method as the reference method. This study is the first to perform a head-to-head comparison of culture report times between conventional and centrifuging and washing methods. We demonstrated that repeated dialysate centrifuging and washing augmented the dialysate sample concentration, shortened culture report times, and increased the culture-positive rate.

Bacterial identification can be achieved using phenotypic-morphology, immune-serology, genotyping, MALDI-TOF-MS, and nanotools [5, 15, 16]. However, all of these methods require an adequate quantity or concentration of bacterial samples to increase the bacterial growth rate in culture plates [17]. The higher the bacterial concentration or quantity, the faster the BACTEC system detects the cultured bacteria resulting in a shorter culture report time. Membrane-adsorption elution, magnetic bead separation, and repeat centrifugation techniques have long been known to increase bacterial concentration. However, these methods’ cost is relatively high, and concentration outcomes are inconsistent [18–20].

In some cases, the increased bacterial concentrations obtained after the C&W method allowed us to observe bacteria directly using light microscopy before the bacterial culture step. Gram stain results were immediately available in these cases. The increased bacterial concentration also allowed us to skip the BACTAC FX system culture step and made it possible to directly plate the bacteria, significantly shortening subsequent report time for pathogen identification and antibiotic susceptibility testing.

Another possible mechanism of accelerated report times may involve the washing procedure, as it may remove inflammatory cells and cytokines that damage bacteria and impede growth [21, 22]. Furthermore, repeated washing with sterile saline increases the pH of the bacterial solution. Given that Dianeal dialysate is a lactate-buffered solution with an unfavorable pH of 5.5 [23], washing with sterile saline may benefit bacterial growth [24]. Using *E. coli* from ATCC, our study showed that repeated washing did not flush out the bacteria and decrease bacterial concentration. However, with the C&W method, we obtained a higher sample ATP concentration and bacterial colony count.

**Fig. 2:**
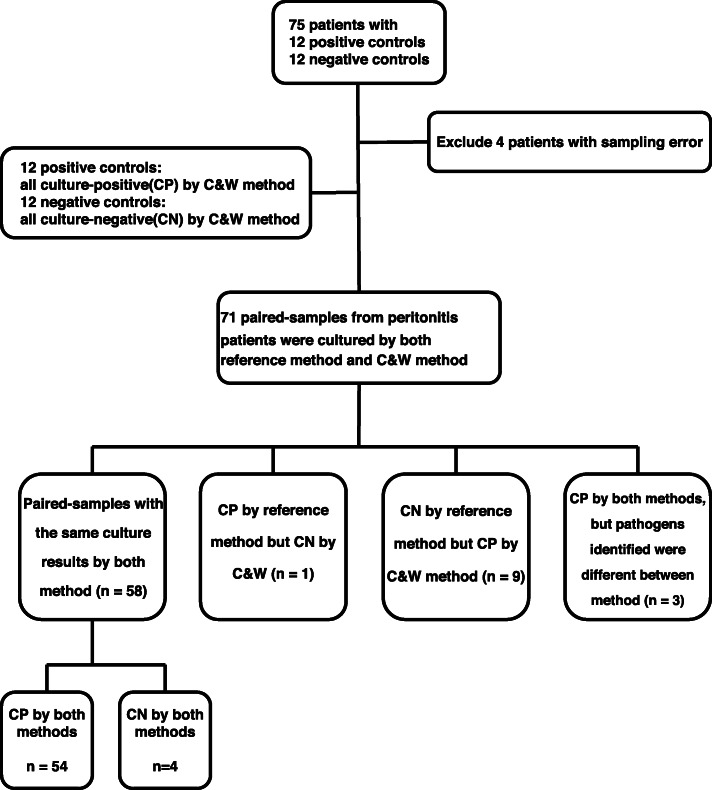
The flow chart of the reference culture method and centrifuging and washing (C&W) method. WBC, white blood cells

Fungal peritonitis was found in three processed cases via the C&W method, but none were identified with the reference method. We used thioglycolate broth for fungal culture, which was added to the supernatants or precipitates in several steps of the C&W method (Fig. 2). Although the chances of detecting fungal infection were not different for the C&W (3/71) and reference methods (0/71) (*p* = 0.245, Fisher’s Exact test) in the current study, early detection of fungal infection could have meant that these three patients had their PD catheter removed earlier.

We used dialysate samples that were large enough to collect adequate bacteria for culture [7]. In a parallel study, we also used the C&W method to process infected cerebral spinal fluid, frequently between 1 mL and 3 mL, or pleural effusion for culture with similarly very positive findings.

As this was a pilot diagnostic accuracy study, we have listed the potential benefits of diagnosing existing pathogens in dialysates faster. We could respond earlier and prescribe the correct antibiotics if results were obtained faster with the C&W culture method. We could also avoid using a nephrotoxic aminoglycoside in Gram-positive infections and prescribe vancomycin earlier in *MRSA* infections. Theoretically, the C&W method could reduce medical costs and duration of hospital stay for many patients.

### Limitations

We had to expose samples to air several times for the C&W method, potentially inhibiting any anaerobic bacteria’s growth. We did not identify any anaerobic bacteria using the reference method, nor did we identify any anaerobic bacteria using the C&W method; therefore, we are uncertain whether anaerobic bacteria will be a limitation. Another potential limitation may be in the identification of mixed flora, frequently seen in urinary tract infections. The predominant bacteria in mixed flora of unprocessed samples may not be as dominant after samples have been centrifuged and washed. The small sample size limits the current study; we only accepted patients who came to the hospital during office hours. We cannot determine whether there will be any differences in the study results if we also accepted patients who came during non-office hours.

## Conclusion

We conclude that sample processing with repeated centrifuging and washing can concentrate bacteria and shorten culture report times. It is a secure and low-cost method. Our pilot study showed that this method could benefit patients by shortening the confirmatory of infection; this would benefit them both physically and economically. However, a prospective randomized control trial using the C&W method results to guide antibiotic treatment is needed to prove this method’s clinical benefits further.

## Methods

### Participants

Patients on peritoneal dialysis (PD) with peritonitis were recruited from China Medical University Hospital. The study protocols were reviewed and approved by the China Medical University & Hospital Research Ethics Committee (reference number: CMUH-REC3–038). All patients signed a written informed consent form before dialysate retrieval. As per the International Society for Peritoneal Dialysis (ISPD) guideline definition, peritonitis was diagnosed in patients whose dialysates had an increase in their white blood cells (WBC) count (> 100/μL) and also had symptoms of abdominal pain or turbid dialysate [7]. We excluded patients with dialysate WBC counts < 100/μL, and patients who had received antibiotic treatment before their dialysate sample was collected. We also excluded any patients who did not come to the hospital during office hours (9 am to 5 pm) to avoid any possible delay in sample processing. Some patients receiving PD who had no abdominal pain and had clear dialysate also donated their dialysate as negative controls.

### Specimen preparation

A total of 40 mL of infected dialysate was retrieved from each patient. The first 10 mL of dialysate was immediately sent for cell count analysis through routine microscopic examination and for routine pre-culture Gram staining. The second and third 10 mL of dialysates were inoculated directly into aerobic and anaerobic BACTEC blood culture bottles (BD, Franklin Lakes, NJ, USA), respectively, for use as the reference dialysate culture. The reference culture method followed the 2010 ISPD recommended guideline [7]. The final 10 mL of dialysate was injected into a sterile Falcon tube (BD) for repeat dialysate centrifuging and washing (C&W method) to increase the dialysate bacterial concentration. The dialysate was immediately sent to the laboratory for culture after its retrieval in a double-blind manner. The person delivered the samples to the laboratory, and the technician in the laboratory did not know the patients’ clinical condition. Following dialysate retrieval, peritonitis patients received cefazolin (1.0 g/kg body weight) and gentamicin (0.5 mg/kg body weight) via the intraperitoneal route immediately and then daily before bedtime.

### Repeat centrifuging and washing method (C&W method)

The detailed procedure of the C&W method is shown in Fig. 2. The same batch or paired-dialysate sample (10 mL) was centrifuged at 700 rpm for 10 min, and the supernatant was aspirated to separate microorganisms from cellular debris. Thioglycolate broth was added to the precipitate (the cellular debris) to prevent any occult bacteria contamination. The supernatant was subsequently centrifuged at 13000 rpm for 10 min. We then added normal saline to the resulting precipitate at a ratio of 5:1 (normal saline: precipitate) and centrifuged the solution for 10 min at 13000 rpm. We removed the supernatant and added normal saline at a ratio of 10:1 to the precipitate and then centrifuged the solution at 13000 rpm for 10 min. Thioglycolate broth was added to the supernatant of all 13,000 rpm centrifuge steps. After repeated centrifuging and washing, the sediments were sent for Gram staining and then inoculated directly into bacterial culture agar plates. The normal saline used was subjected to bacterial culture to avoid possible contamination during the C&W procedure.

### The reference method (conventional method)

The detailed procedure of the conventional method is shown in Fig. 2. The dialysate samples (10 mL) were inoculated into aerobic and anaerobic blood culture bottles and incubated directly in the BACTEC FX blood culture system (BD), with continuous fluorescent monitoring. This culture method served as the reference method in the study. When bacterial signals were detected, a Gram staining study was performed, and the samples were streaked onto different agar plates, such as EMB/BP and Chocolate agar (BD) for different cultures [25]. A Phoenix Automated Microbiology System, combined with MIC/ID antimicrobial susceptibility kits (BD), was used for microbial identification and antibiotic susceptibility testing. The study was performed following the manufacturer’s recommendations [25]. Patients were taken off gentamicin if the culture report showed Gram-positive bacterial. If the culture results showed *methicillin-resistant staphylococcus*, the vancomycin antibiotic was administered to the relevant patient.

### Supplementary method for bacterial identification

Bacterial colonies isolated from agar plates were analyzed through MALDI-TOF MS using a short pulse laser (Bruker Daltonics) [3]. The resulting spectra were analyzed using built-in Practical Atlas for Bacterial Identification software [26]. MALDI-TOF-MS served as a supplementary method for microbial identification.

### Bacterial mass measurement

The presence of ATP in living bacterial cells can be used to estimate bacterial mass [27]. The ATP generation test was applied for bacterial mass measurement. Commercial ATP generation test kits (3 M Clean-Trace Water Test, St Paul, Minnesota, USA) were used to estimate and compare the dialysate concentration with and without C&W treatment. *Escherichia coli* (American Type Culture Collection (ATCC), 25,922) and *Staphylococcus aureus* (ATCC 25923) was used as a positive control group. The ATP-free swab was briefly dipped into the *E. coli* (1 × 10^4^ CFU/mL) solution with and without C&W treatment. The swab was then placed into an ATP-free solution containing D-luciferin, and the test swab was placed into a Clean-Trace ND luminometer (3 M) for measurement [28].

### Data analysis

The culture results obtained by both methods were reported once pathogens were identified, and as soon as antibiotic susceptibility test results become available. The report times for each sample and method were recorded. When the BACTEC culture system failed to detect bacterial signals in the culture bottles after six days of culture, the results were reported as culture negative. Variables from both groups were compared by t-test when the data were presented as mean ± standard deviation and passed the normality test. The Mann-Whitney test was used when data were presented as the median (interquartile range) and failed to pass the normality test. A *p*-value of < 0.05 was regarded as being statistically significant.

## Data Availability

The datasets used and analyzed during the current study are available from the corresponding author upon request.

## Abbreviations

PD: Peritoneal dialysis
C&W Method: Centrifuging and washing method
MALDI-TOF MS: Matrix-assisted laser desorption/ionization time-of-flight mass spectrometry
ATCC: American Type Culture Collection
*E. coli*: *Escherichia coli*
*S. aureus*: *Staphylococcus aureus*
CFU: Colony-forming units
IQR: Interquartile range
RLU: Relative light units
WBC: White blood cells
CP: Culture-positive
CN: Culture-negative
*MRSA*: *Methicillin-resistant Staphylococcus aureus*
TBS: Tryptic soy broth
ISPD: International Society for Peritoneal Dialysis
